# Adolescent agency and behavioral characteristics: conformity, problematic behavior, need for cognition

**DOI:** 10.3389/fpsyg.2025.1410170

**Published:** 2025-04-03

**Authors:** Mikhail Goshin, Dmitry Grigoryev, Pavel Sorokin

**Affiliations:** ^1^Pinsky center of General and Extracurricular Education, Institute of Education, National Research University Higher School of Economics, Moscow, Russia; ^2^Center for Socio Cultural Research, National Research University Higher School of Economics, Moscow, Russia; ^3^Laboratory for Human Capital and Education Research, Institute of Education, National Research University Higher School of Economics, Moscow, Russia

**Keywords:** agency, behavioral characteristics of adolescents, conformity, problematic behavior, need for cognition

## Abstract

The concept of human agency has attracted considerable interest in academic and expert discussions concerning various life domains and age groups, including adolescents. However, the field lacks a unified approach to measuring agency for adolescents and understanding its relationship with their behavioral characteristics and attitudes. This study addresses these gaps by introducing an Adolescent Agency Index and examining its associations with behavioral characteristics such as сonformity, problematic behavior, and the need for cognition. The data was collected from students in grades 4 to 8 (*N* = 4,603; *M_age_* = 12.6, *SD* = 1.7; 50.3% female). The analyses indicated a negative relationship between agency and conformity and a positive relationship between agency and problematic behavior; moreover, a stronger positive relationship was found with the need for cognition. At the same time, the nature of the relationship between agency and these characteristics is complex, non-linear. These findings provide valuable insights for educational policymakers, researchers, and families.

## Introduction

The rapidly changing reality brings new challenges to various social and demographic groups (including teenagers) that require the ability for an independent individual action. Such problems as climate change, economic instability and inequality, human rights violations, military-political conflicts, and pandemics require the transformative action on individual as well as organizational and institutional level (Manyukhina, 2022). In this context, formation of agency, that is, the ability to make decisions and take initiative in various contexts and spheres of public and personal life relying primarily on yourself (and not on the external control or support) is becoming increasingly important for education policy (Sorokin and Froumin, 2022). No less important is understanding the fundamental principles of how agency evolves and manifests itself in the highly diverse and complex social environments (Archer, 2024).

### Defining agency

Agency is generally defined as the ability of an individual to act (Gao, 2010), exercising control over his/her life, the ability to set and achieve goals (Cavazzoni et al., 2021a), proactively influence the environment, including the transformation of existing and the creation of new forms of interaction in various spheres of public life (Udehn, 2002; Sorokin and Froumin, 2022). Agency is expressed in the ability to be an active participant, guiding and shaping one’s life path and surrounding contexts (Schoon and Cook, 2021). It also implies the optimization of resources, overcoming or transforming constraints on the way to achieve self-set goals (Zimmerman and Cleary, 2006), both in individual subjective reality and in objective social reality. Literature (Virkkunen, 2006; Hopwood and Sannino, 2023) also notes that the key aspect of agency is the idea of struggle which invokes going beyond, breaking away, transcending the status quo. There are a variety of approaches to the definition of agency as well as difficulties in searching for the optimal methodological solutions for measuring this construct, which are primarily due to the multidimensional (umbrella) character of this concept (Schoon and Cook, 2021; Schoon and Heckhausen, 2019). The increase in interest to agency concept across international academic and expert discussions in recent years can be explained by the accelerating uncertainty and turbulence. Growing is recognition of the necessity for searching for the new sources of positive social transformations in the context when previous structural factors of development (globalization and international cooperation, expansion of education, markets and democratic institutions, etc.) - are almost exhausted. Neo-structuration has been proposed as a term for defining a new phase of societal evolution when social institutions and structures across different domains of life become increasingly dependent upon individual agency (Sorokin and Mironenko, 2025). This challenge makes vital the task of measuring human agency - especially in such social and demographic groups that remain a priority for social and education policy, including adolescents.

### Creating opportunities: agency as a driver of change

Sociological literature as well as common sense and dominant cultural narratives suggest that people have the ability to act strategically carrying out purposeful actions that lead to changes in the external world (Oswell, 2013; Archer, 2013; Sorokin, 2020; Mironenko and Sorokin, 2022). As recent review shows, the individual ability for transformative action is based on two components (Cavazzoni et al., 2021a). On the one hand, there are personal characteristics, such as “self-efficacy,” “personal autonomy,” “optimism,” “self-esteem,” “internal locus of control,” “self-identification boundaries” (Bazzani, 2023; Hitlin and Kwon, 2016), and others. On the other hand, the manifestation of these qualities is dependent upon the structural capabilities. For example, socio-economic conditions, the territory of residence, family, ecological environment, etc. Both components are in dialectical interaction: in the absence of structural conditions, individual potential is difficult to unleash, while even the best structural capabilities are useless without individuals with intentions and the ability to act (Manyukhina, 2022; Abebe, 2019; Veronese et al., 2019). Since the 1990s sociologists have struggled to integrate structure and action theories, focusing on individuals’ capacity to act within societal constraints (Mick, 2021). For instance, the Capability Approach offers a framework for understanding the dynamic relationship between agency and structure, emphasizing the role of conversion processes in transforming resources into valued outcomes. These processes are influenced by individual characteristics and multi-layered structural factors, highlighting the interplay between personal agency and societal conditions (Hvinden and Halvorsen, 2018). This approach provides insights into how individuals, such as persons with disabilities, can combat exclusion and achieve equal participation in society. However, the limitation of this approach is insufficient attention to such a type of individual action that is capable of not only using existing structural opportunities (Mironenko and Sorokin, 2022; Sorokin, 2020), but also creating new ones. This limitation is typical for the vast majority of research on agency (including in psychology), as recent reviews show (Cavazzoni et al., 2021a; Cavazzoni et al., 2021b; Mironenko and Sorokin, 2022), including research on agency in educational environments. For example, J. Reeve et al. (2020) in a highly cited work utilize a tool for measuring adolescents’ “Agentic engagement,” which focuses exclusively on the behavior during classes and mostly on communication with teacher (“I let my teacher know what I need and want,” “I let my teacher know what I am interested in,” “I express my preferences and opinions,” etc). But beyond the scope are the social/structural results of these communication: what is the impact of expressed opinion? How are decisions made about the actual educational activities? Is the student’s voice heard and to what extent is it taken into account by the teacher? Another limitation is that, in general, existing literature on adolescence agency focuses primarily on education sphere and family (Cavazzoni et al., 2021a), while such aspects as relations with peers and earning money - appear understudied.

### Adolescence and the formation of agency

Adolescence is a critical period for developing agency, which plays a crucial role in identity formation, life satisfaction, and the ability to navigate challenges and opportunities in both personal and social contexts (Morsunbul, 2013; Vats and Biswas, 2024). Studies have shown the link between agency (understood as proactive behavior) and the psychological well-being of adolescents and their more successful social adaptation (Gallagher et al., 2019). The rapid physical, cognitive, and emotional development that occurs in adolescence allows young people to achieve greater autonomy from their parents, understand their interests and goals in professional and personal life, resulting in the expansion of their “horizon of possibilities.” According to the existing literature, an important manifestation of agency is the participation of adolescents in making decisions that affect their lives (Thoits, 2006). It is the ability to make decisions in various circumstances of a changing life that allows young people to feel fully in charge of constructing their life path and their social reality, especially, in times of neo-structuration, when social environment transforms rapidly and individual agency becomes more in demand than ever before (Mironenko and Sorokin, 2022).

### Agency and behavioral characteristics in adolescents

Agency, as a characteristic, dealing with orientation towards change, may be expected to negatively correlate with *conformity*. However, literature shows that agency and conformity are in a complex relationship. Conformity is an individual’s ability to accept the positions, attitudes, or behavior of the group to which they belong or the one towards which they are oriented (Coultas and Van Leeuwen, 2015; Wijenayake et al., 2020). Conformity can manifest itself in various forms: from acceptance of peer opinion to submitting to social norms, which can sometimes lead to suppression of individuality and originality (Rahmatika and Kusmaryani, 2020). Since agency implies transforming existing social structure (at least, to a certain extent), high levels of schoolchildren’s agency may be negatively related to conformity (Kirby, 2019; Sorokin and Froumin, 2022; Virkkunen, 2006; Hopwood and Sannino, 2023). That is, if students feel they can control their own actions and have confidence in their decisions, they are less likely to succumb to pressure from others. For instance, they can express their thoughts and ideas, even if they do not agree with the group’s opinion.

On the other hand, research on conformity and agency reveals complex relationships influenced by cultural and individual factors (Güngör et al., 2014). Thus, in an East Asian cultural context conformity can be seen as a component of so-called “interdependent agency,” while in Western cultures conformity is understood as a lack of autonomy and agency (Kagitcibasi, 2005). Literature suggests that, in some cases, conformity may contribute to the manifestations of agency. Group membership and fulfillment of social roles in some circumstances may require a high degree of individual agency (Bazzani, 2023; Choi et al., 2019). Sarab Abu-Rabia-Queder (2008) note that the use of conformist behavior by Middle Eastern feminists (e.g., veiling) contributes to further social change. If students believe that in order to achieve their goals they need to integrate into a certain group, they may exhibit behavior that conforms to the expectations of that group (Rahmatika and Kusmaryani, 2020; Imansyah and Setyawan, 2018). It is important to note here that students may use conformity as a strategy to adapt to the social environment to avoid conflicts or to gain peer approval. It is noted (Imansyah and Setyawan, 2018) that while moderate conformity may aid adaptation to school environments, excessive pressure can reduce internal motivation and academic performance.

Thus, agency and conformity are in a dynamic interaction: adolescents, possessing a certain degree of agency, can either resist conforming behavior or selectively integrate it into their actions, purposefully managing their behavior depending on the situation.

The second aspect, to some extent the opposite of conformity, is *problematic behavior*. Research reveals complex relationships between self-focus, interpersonal dynamics, and behavioral outcomes. Helgeson and Fritz (2000) report that problematic behavior is primarily associated with “unmitigated agency,” that is, a form of agency that implies the absolute focus of the subject on the “self,” without taking into account the interests, opinions and needs of others. The review (Cavazzoni et al., 2021b) notes that ambiguous, aggressive, or dangerous behavior in adolescents can manifest itself as a form of agency to provide protection and safety in difficult social situations. The concept of “ambiguous agency” (Bordonaro and Payne, 2012, p. 366) is an important step towards understanding that part of agentic behavior, which stands in contrast to a definition of agency purely defined by a set of constructive and positive actions. “Аmbiguous agency” implies patterns of behavior that can be described as deviant or inconvenient, but which, nevertheless, can be crucial for the survival of adolescents and the improvement of their living conditions (Bordonaro and Payne, 2012).

Moreover, going beyond existing structural frameworks (which is implied by agency) may, in some cases, result in behavior that transcends typical social norms and expectations, thereby triggering changes in the dynamics of interactions between people (Kamphaus, 2010; Beam et al., 2002; Padilla-Walker et al., 2018). Agency, the capacity to influence one’s environment, can shape, interpret, and manage norms (Angstadt and Möller, 2020). This interaction is crucial in social tipping processes, where nonlinear transformations improve sustainability and well-being (Gaupp et al., 2023). However, in general, the relationship between these phenomena has not yet been well-studied empirically.

*Need for cognition (NFC)* is another psychological construct which might be associated with agency (Cacioppo and Petty, 1982). NFC reflects an individual’s tendency to seek, engage in, and enjoy effortful cognitive activities (Jebb et al., 2016; Lavrijsen et al., 2023). The agency of adolescents was claimed to be connected with educational aspirations (Schoon, 2006) and school engagement (Schoon, 2008). In the article (Jebb et al., 2016) it is shown that NFC is positively associated with innovative behavior. While NFC correlates with openness to experience, research has shown that it not just positively related to academic performance (Lavrijsen et al., 2023), but also demonstrates incremental validity in predicting goal-oriented behavior (Fleischhauer et al., 2010), as well as moderates the relationship between adequately challenging schoolwork and students’ intrinsic motivation and engagement, with high-NFC students benefiting more from challenging tasks (Lavrijsen et al., 2021).

Accordingly, agency may require a high NFC, which may also be seen in terms of manifestation of reflexivity understood from sociological perspective (see in more detail the argumentation from the standpoint of sociological theory in the works of Emirbayer and Mische, 1998; Archer, 2013). Such a relationship has also not been investigated sufficiently.

### Diverse contexts of adolescent agency

An important feature of agency is its multi-contextual character: humans manifest their proactive activity transforming social context in various fields which have not yet been analyzed systematically (Cavazzoni et al., 2021a). This is obviously true for adolescents who exhibit agency in different ways in various contexts. For example, three different spheres of agency manifestation have been outlined in literature but not related to each other empirically, i.e., with parents, teachers, and peers (Gurdal and Sorbring, 2018). Previous studies show that the manner in which adolescents manifest their agency may vary. When dealing with adults such as parents or teachers, agency may be demonstrated by ignoring or refusing. When interacting with peers, children tend to employ democratic solutions to express their agency. The existing studies of adolescence’ agency have a number of significant limitations: firstly, the majority of studies (see, for instance, Gurdal and Sorbring) is based on interviews and does not involve the development of an instrument for quantitative analysis, in particular, it does not set the task of developing a holistic agency index. Secondly, the instruments proposed by the authors generally rely mostly on fictional situations that are offered to the respondent, while the analysis of the respondent’s real experience in terms of the manifestation of agency (including not only the action itself, but also its objective structural impact) is very limited or not performed at all (Reeve et al., 2020). Finally, the existing frameworks used for the empirical analysis leave out such an important aspect of a teenager’s agency as self-initiated activity in the field of earning money, which becomes an increasingly relevant as recent studies show (see Staff et al., 2023).

## Current study

The concept of agency, despite increasing scholarly and expert interest, lacks a consensus on assessment methodologies (Cavazzoni et al., 2021a). Agency’s multifaceted nature means it can manifest differently across various domains, leading to a demand for universal models for analyzing and interpreting empirical findings related to its diverse expressions simultaneously (Gurdal and Sorbring, 2018). Addressing this gap, our study introduces a novel tool designed to assess adolescent agency, focusing on four key areas of manifestation: interactions with parents, peer relationships, choice of leisure activities, and money-earning endeavors. The selection of these domains is grounded in a review of extensive empirical evidence and theoretical discussions surrounding adolescent agency issues as outlined in prior research (Gurdal and Sorbring, 2018; Nunes et al., 2023; Staff et al., 2023) analyzed above.

This study aims to investigate the relationship between adolescents agency and their several behavioral characteristics, based on the proposed assessment tool. Despite the existing examples of integration of certain aspects of conformism into behavior designed to become agentic (Güngör et al., 2014; Abu-Rabia-Queder, 2008), especially in East Asian cultures (“interdependent agency”), these manifestations can be considered rather as strategies of adaptation to the social environment with the aim of its subsequent transformation. Agency implies the capacity to innovate and create new communities, practices and forms of social interaction, which exceed existing norms. Thus, agency is hypothesized to be contrasting with conformity (Kirby, 2019). With this framework in mind, we expect a negative correlation between adolescents’ levels of conformity and their expression of agency (H1).

Problematic behaviors can be part of such forms of agency as “unmitigated agency” (Helgeson and Fritz, 2000) and “ambiguous agency” (Bordonaro and Payne, 2012), and transformation of social structures may require protest behavioral manifestations, drawing support from the existing literature (Neale et al., 2022; Kamphaus, 2010; Beam et al., 2002; Padilla-Walker et al., 2018; Hopwood and Sannino, 2023). Accordingly, we assume a positive correlation of adolescents agency with behaviors of protest or problematic behaviors that challenge existing social structures (H2).

Finally, NFC shows a positive relationship with educational aspirations (Schoon, 2006), school engagement (Schoon, 2008), innovativeness (Jebb et al., 2016), openness to new experiences (Lavrijsen et al., 2023) and goal setting (Fleischhauer et al., 2010). Accordingly, we hypothesize a strong positive relationship between NFC and agency (H3).

## Method

### Participants

The study is based on the results of a survey of students in grades 4 to 8 (*N* = 4,603; *M_age_* = 12.4, *SD* = 1.46; 50.3% female) conducted in 2022 in Yaroslavl, Russia. The population of Yaroslavl is about 600,000 people. The sample is representative of urban schools: a random stratified selection of schools was carried out, taking into account their size as a characteristic of the socio-economic status of an educational organization. A total of 31 schools were selected for the study. The structure of the sample of respondents is presented in Table 1.

**Table 1:** The structure of the sample of respondents.

Characteristics	*N*
The family’s characteristics (or people, living together with an adolescent in the same household)	
Mother	91%, *N* = 4,202
Father	73%, *N* = 3,357
Grandparents	16%, *N* = 718
Siblings	49%, *N* = 2,250
Other relatives	8%, *N* = 385
The level of education of the mother	
General secondary education or lower	10%, *N* = 472
Elementary or secondary vocational education	41%, *N* = 1855
Tertiary professional education	49%, *N* = 2,239
The level of education of the father	
General secondary education or lower	13%, *N* = 573
Elementary or secondary vocational education	47%, *N* = 2028
Tertiary professional education	40%, *N* = 1739
The grade in which the respondent studies	
4th	19%, *N* = 881
5th	20%, *N* = 905
6th	21%, *N* = 979
7th	21%, *N* = 957
8th	19%, *N* = 881

### Procedure

Before conducting the study, the organizers asked the parents to give their consent for the participation of their children in the study. The study involved those adolescents whose parents filled out informed consent. The survey took place within the framework of class hours and in schools’ computer classrooms. The questionnaires were completed online on the Alchemer platform^1^. Each participant was assigned a unique identification number. The duration of filling out the questionnaires did not exceed 40 min. A supervising teacher was present in the classroom during the entire time of the survey.

### Measures

#### Agency index

**Figure 1:**
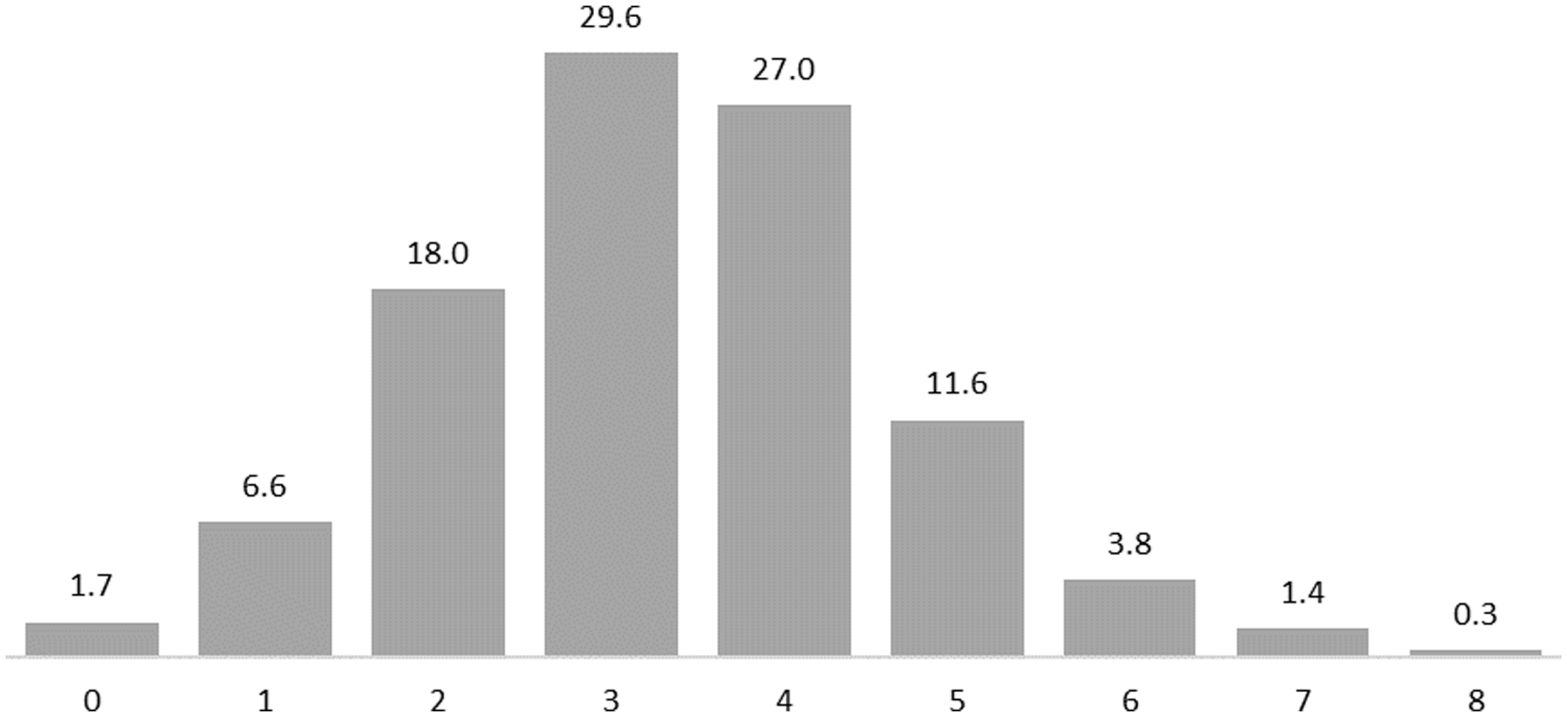
Frequency distribution of AAI, in percentages (%).

To measure the multi-faceted construct of ‘Adolescents Agency,’ our method employs a composite “Adolescents Agency Index,” (AAI) inspired by established metrics for complex formative constructs like ‘well-being’ and ‘quality of life’ (Michalos, 2014). This index aggregates data from four key domains: family decision-making, peer interactions, leisure activities, and activities related to the financial field. The AAI was computed using responses to four targeted questions, each probing different aspects of agency manifestation. These questions addressed *decision-making in family settings* (“How are decisions made about joint activities with your parents?”), *peer interactions* (“How do you and your friends typically decide what activities to undertake together?”), *leisure choices* (“To what extent do your parents influence your choice of leisure activities?”), and *money-earning endeavors* (“What is your primary source for personal expenses?”). Agency was quantified on a scale from 0 to 2 points based on respondents’ answers to these questions. A score of 0 was assigned for the absence of agency, as evidenced by responses like “I do not participate in decision-making” or “parents suggest - I agree.” A score of 2 indicated a clear demonstration of agency, with responses such as “most often I take the initiative and friends support me” or “I choose leisure by myself only.” Intermediate agency levels were assigned a score of 1, characterized by responses like “my parents helped me in choosing leisure” or “I usually support the initiative of my friends after we discuss it.” The resulting AAI ranged from 0 to 8 points, with a mean of 3.32 (*SD* = 1.37; *Md/Mode* = 3.00). The distribution of the scores was approximately normal, as indicated by a skewness of 0.19 and kurtosis of 0.31 (see Figure 1).

The participants were also classified into distinct agency categories based on their scores: those with scores of 0 or 1 were categorized as ‘Minimum Agency’; a score of 2 was labeled ‘Low Agency’; a score of 3 was termed ‘Slightly Lower Agency’; a score of 4 was described as ‘Slightly Higher Agency’; a score of 5 was dubbed ‘High Agency’; and scores ranging from 6 to 8 were grouped under ‘Maximum Agency.’

#### Behavioral characteristics of adolescents

In addition, we applied principal component analysis to estimate each of three core adolescent behavioral characteristics (Table 2). These characteristics were: (1) *Conformity*, inspired by Uchida et al. (2020), and assessed by respondents’ agreement with the following statements “Parents and teachers say that it is very important to study, so I study and go to school,” “I have to go to school and fulfill all the requirements of teachers,” and “I solve examples only as the teacher says”; (2) *Problematic Behavior*, informed by Kamphaus (2010), gauged through the level of agreement with the next statements “I usually do not do homework,” “A disrupted lesson can be entertaining,” and “I sometimes skip classes”; (3) *Need for Cognition*, inspired by Cacioppo and Petty (1982), evaluated through the degree of agreement with statements “If I did not understand something in the lesson, I will definitely try to figure it out,” “When we go through a new topic, I want to figure out incomprehensible questions,” and “I like to cope with difficult tasks,” having a Cronbach’s alpha of 0.64 and component loadings from 0.653 to 0.776.

**Table 2:** Behavioral characteristics of adolescents.

	*M*	*SD*	Cronbach’s alpha	Component loadings ranging
Conformity	9.46	2.05	0.55	0.533–0.757
Problematic Behavior	4.80	1.97	0.53	0.511–0.796
Need for Cognition	8.94	2.27	0.64	0.653–0.776

min.–max. = 0–12, for each characteristic.

For each of the parameters (Conformity, Problematic Behavior, NFC) the respondent could score from 1 to 12 points. Accordingly, like the distribution by levels of Adolescents Agency, the respondents were separated into 4 groups (levels) for each parameter, based on the number of points scored as follows: the first level (minimum expression of the parameter) - from 1 to 3 points; the second level - from 4 to 6 points; the third level - from 7 to 9 points; the fourth level (maximum expression of the parameter) - from 10 to 12 points.

## Results

In a linear ordinary least squares regression analysis focusing on how adolescents’ behavioral characteristics predict their levels of agency, as measured by the AAI, the model explained 5% of the variance, *F*(3, 4,599) = 85.5, *p* < 0.001:


$$\begin{array}{l} AAI=1.92–0.07\times \mathrm{Conformity}\\ \;+0.05\times \mathrm{Problematic}\;\mathrm{Behavior}\\ \;+0.15\times \mathrm{Need}\;for\;\mathrm{Cognition}+\in \text{.} \end{array}$$

Within this analysis, Conformity was negatively associated with agency, indicating that higher levels of conformity are linked to lower levels of agency (H1; Table 3). Problematic Behavior was also positively related to agency, though to a lesser extent (H2). On the other hand, the NFC emerged as the strongest predictor, showing a significant positive relationship with the AAI (H3).

**Table 3:** Linear ordinary least squares regression analysis focusing on how adolescents’ behavioral characteristics predict their levels of agency.

	*β*	*SE*	*t*	*p*
Conformity	−0.11	0.01	−6.8	<0.001
Problematic behavior	0.07	0.01	4.7	<0.001
Need for cognition	0.26	0.01	15.8	<0.001

*R*^2^ = 0.05, *F*(3, 4599) = 85.5, *p* < 0.001.

**Figure 2:**
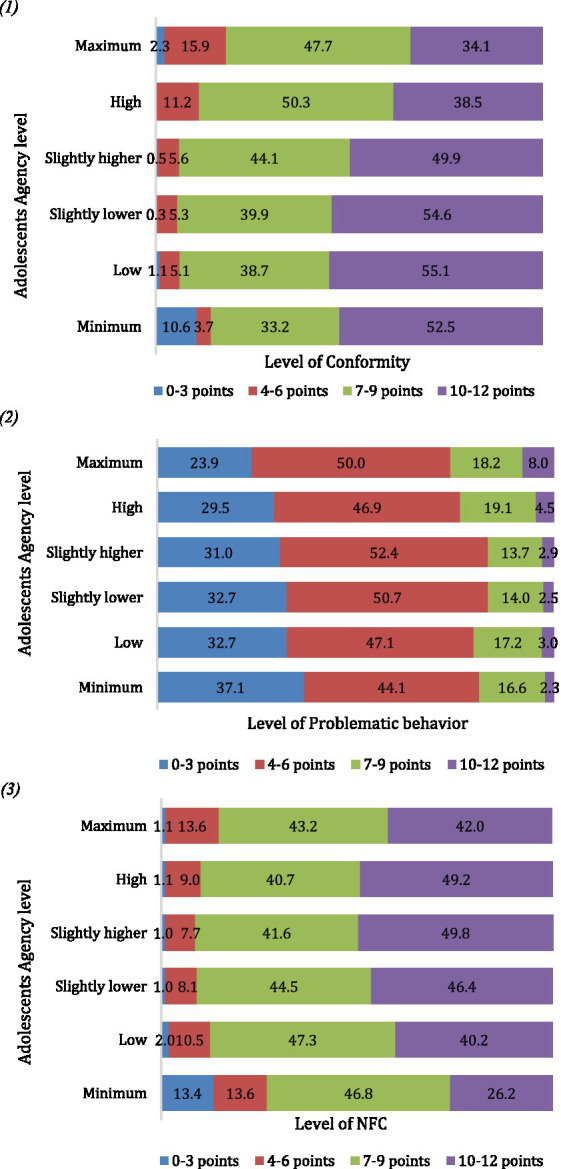
The relationship between the adolescents agency level, and the conformity (1), problematic behavior (2), and NFC (3), in percentages (%). (1) χ^2^(df) = 395.853 (15), *p* < 0.001; (2) χ^2^(df) = 40.154 (15), *p* < 0.001; (3) χ^2^(df) = 403.980 (15), *p* < 0.001.

However, subsequent crosstabs analysis between levels of adolescents agency and levels of expression of the studied behavioral characteristics of adolescents revealed a complex, non-linear relationship between these behavioral characteristics and agency levels. Regarding Conformity, it showed a slight increase when moving from minimum to low levels of agency. However, beyond these levels, as agency increased, Conformity decreased, suggesting that higher agency is associated with lower levels of Conformity (Figure 2 (1)). In contrast, the link between Problematic Behavior and agency indicated a direct relationship: higher levels of agency were associated with increased likelihoods of engaging in Problematic Behavior (Figure 2 (2)). Specifically, the NFC was lowest at the lowest levels of agency, peaked at moderate levels of agency, and decreased slightly at the highest levels of agency, though still higher than at the lowest level (Figure 2 (3)).

## Discussion

This research employed a novel approach to create an integrative index measuring adolescent agency, focusing on its expression in four key domains: interactions with parents, peer relationships, leisure activity choices, and financial activities. These areas were selected based on previous studies (Gurdal and Sorbring, 2018; Nunes et al., 2023; Staff et al., 2023), which highlighted their significance as potential contexts for the demonstration of agency (however, with an introduction of a financial field as a distinct dimension for agency manifestation, which was ignored by previous literature). The present study explored how this adolescents agency index (levels) associates with selected adolescent behavioral characteristics, including conformity, problematic behavior, and the need for cognition.

### Agency and conformity (H1)

The analysis revealed a nuanced relationship between adolescents’ levels of agency and their tendency towards conformity. Generally, a slight negative correlation exists, indicating that higher agency levels often lead to less conformity, and this is consistent with available literature (Schwartz et al., 2005). However, this trend reverses for those with agency levels at or below the average, where agency positively correlates with conformity. This finding partially supports H1. Such results align with a more refined view of agency found in the literature, which recognizes children’s creative capabilities and moves beyond the simplistic view of agency merely as resistance to conformity (Bazzani, 2023; Kirby, 2019; Sorokin and Froumin, 2022; Sorokin, 2020). This perspective suggests that, in some contexts, conforming to social norms can itself be an act of agency - for instance, in the context of neo-structuration, when structural conditions are changing and innovations are necessary in order to support existing order of relations and practices (Rahmatika and Kusmaryani, 2020; Taylor et al., 2019; Imansyah and Setyawan, 2018; Sorokin and Mironenko, 2025). These findings suggest that agency is a nuanced concept that interacts with social norms and structural factors in context-specific ways. Understanding these interactions is crucial for developing effective interventions to promote adolescent well-being (Taylor et al., 2019). This study is pioneering in showing the potential relevance of positive aspects of agency, including the capability for social norm compliance. In contrast, higher agency levels, associated with active efforts to reshape social reality (Udehn, 2002), correlate with a reduced inclination towards conformity - as initially expected.

### Agency and problematic behavior (H2)

The observed positive link between adolescent agency level and problematic behavior can be interpreted in the following way: agency involves challenging existing norms and patterns, taking proactive, independent actions, and fostering innovation (Cavazzoni et al., 2021a; Cavazzoni et al., 2021b; Gao, 2010; Hopwood and Sannino, 2023). Moreover, as the authors (Neale et al., 2022) point out, deviance during adolescence can promote the development of and reliance upon relational capital; these characteristics are associated with innovativeness, proactiveness, and risk-taking as adults. This supports H2. Yet, the modest strength of this relationship suggests that agency cannot be simply equated with rebellious behavior; it is a more intricate construct (refer to Sorokin and Froumin, 2022; Sorokin, 2020 for a more in-depth discussion). This finding underscores the complexity of agency, highlighting its role in both constructive innovation and in behaviors that may be deemed problematic, without reducing it to mere oppositional conduct.

### Agency and NFC (H3)

Interestingly, the relationship between the NFC and adolescents agency levels is most pronounced at average levels of agency, forming a U-shaped pattern. This indicates that a high need for cognition does not necessarily predict high levels of agency, thus, partially supporting H3. This observation suggests that while the NFC is an essential aspect of agency (Lavrijsen et al., 2023; Jebb et al., 2016; Schoon, 2006, 2008), agency encompasses a broader range of characteristics and capacities. This complexity hints at the multifaceted nature of agency, which extends beyond cognitive inclinations to include various other dimensions–first of, behavioral characteristics.

### Limitations and future research

This article presents the initial findings from an empirical study on adolescents agency understood as proactive behavior in various social contexts based on personal initiative and implying activities going beyond merely following the external structural requirements or expectations but aimed at transforming or proactively supporting surrounding world. We utilized a newly devised tool to calculate an integral agency index, which allowed indicating different levels of agency. However, the study faces certain limitations, including the restricted age range of participants and its confinement to a single city, limiting the exploration of broader structural and cultural context effects. Moreover, the novel tool does not fully capture all potential areas and motivations behind adolescents’ agency. It was not considered whose interests were pursued by persons when performing an agent’s action, whether it was easy for them to achieve the goal, whether planning and subsequent reflection of their actions were carried out. The study’s correlational design also precludes establishing causality, highlighting the need for future longitudinal research.

Efforts will be made in upcoming studies to address these limitations. Specifically, plans include developing a more detailed measure for assessing adolescents’ agency across various domains such as various aspects of education, family and peer interactions, and economic activities, with a focus on understanding their goals and whether they lean towards personal or communal well-being. In addition, to address existing limitations, it is planned to use longitudinal designs, more diverse samples with wider geographical coverage, and the incorporation of mixed-method approaches that can enhance the richness and applicability of the research findings.

### Theoretical and practical value of the study

Under the current global crisis, when previously dominant structural factors of development (for instance, markets’ and international cooperation’ expansion in late 20th and early 21th century) are exhausted, human agency is seen as one of important potential driving forces of positive social change (Hopwood and Sannino, 2023). Neo-structuration (the current stage of societal evolution) implies that even the most authoritative, solid and efficient social structures (including families, corporations, communities, states, etc.) become dependent upon individual and collective action, transforming environments, producing innovations, thus, supporting both individual and collective well-being (Sorokin and Mironenko, 2025). In this context, enhancing individual agency is an urgent task for practical education policy, including when dealing with adolescents (Sorokin and Froumin, 2022). However, the tools for measuring adolescents’ agency are lacking (Cavazzoni et al., 2021a). Most importantly, required are such instruments that take into account simultaneously several fields of agency manifestation and make emphasis on the actual experience of agentic behavior in the related areas, including its “structural” impact. That means, for instance, analyzing the general practice of making decisions in family, among peers, and in other contexts with the focus on the potential of an adolescent to influence the final decision, and not only the fact that an “opinion” had been “expressed” (see Reeve et al., 2020). Additional research is needed to gain deeper comprehension of the relations between different aspects of agency (for instance, between the “projective” and “practical-evaluative” components; Emirbayer and Mische, 1998) as well as between manifestations of agency in various subject fields. The present paper contributes to this work by introducing the integral AAI and demonstrating complex and dynamic interrelations between agency and Conformity, Problematic Behavior and NFC. The non-linear nature of these interrelations confirm the relevance of AAI as a separate parameter, not reducible to other characteristics, having more universally accepted measurement tools. This has not only practical but also theoretical importance: the Adolescents’ Agency measurement tool may stimulate elaboration of novel theoretical concepts and models of personality and personality development, including in youth studies. In particular, promising may be creating complex models of individual behavior, integrating data on activities in different contexts or subject fields.

## Conclusion

This study introduces an Adolescent Agency index and examines its associations with behavioral characteristics and attitudes of adolescents. The research found that adolescent agency is most strongly correlated with the need for cognition and shows a less pronounced, yet positive, relationship with problematic behavior. Generally, agency and conformity are inversely related, but the interactions with conformity, and particularly with the need for cognition, exhibit a complex, non-linear pattern. Agency might be also related to other personal characteristics, as well as structural contexts, including factors of the school environment and features of interaction in the family. Future studies will be devoted to the study of these relationships.

## Data Availability

The data analyzed in this study is subject to the following licenses/restrictions: The datasets generated and/or analyzed during the current study are not publicly available but are available from the corresponding author on reasonable request. Requests to access these datasets should be directed to Mikhail Goshin, m.goshin@mail.ru.

## Footnotes

^1^ https://www.alchemer.com/
